# Red Queen Processes Drive Positive Selection on Major Histocompatibility Complex (MHC) Genes

**DOI:** 10.1371/journal.pcbi.1004627

**Published:** 2015-11-24

**Authors:** Maciej Jan Ejsmond, Jacek Radwan

**Affiliations:** 1 Institute of Environmental Sciences, Jagiellonian University, Kraków, Poland; 2 Department of Arctic Biology, The University Centre in Svalbard, Svalbard, Norway; 3 Evolutionary Biology Group, Faculty of Biology, Adam Mickiewicz University, Poznań, Poland; La Jolla Institute for Allergy and Immunology, UNITED STATES

## Abstract

Major Histocompatibility Complex (MHC) genes code for proteins involved in the incitation of the adaptive immune response in vertebrates, which is achieved through binding oligopeptides (antigens) of pathogenic origin. Across vertebrate species, substitutions of amino acids at sites responsible for the specificity of antigen binding (ABS) are positively selected. This is attributed to pathogen-driven balancing selection, which is also thought to maintain the high polymorphism of MHC genes, and to cause the sharing of allelic lineages between species. However, the nature of this selection remains controversial. We used individual-based computer simulations to investigate the roles of two phenomena capable of maintaining MHC polymorphism: heterozygote advantage and host-pathogen arms race (Red Queen process). Our simulations revealed that levels of MHC polymorphism were high and driven mostly by the Red Queen process at a high pathogen mutation rate, but were low and driven mostly by heterozygote advantage when the pathogen mutation rate was low. We found that novel mutations at ABSs are strongly favored by the Red Queen process, but not by heterozygote advantage, regardless of the pathogen mutation rate. However, while the strong advantage of novel alleles increased the allele turnover rate, under a high pathogen mutation rate, allelic lineages persisted for a comparable length of time under Red Queen and under heterozygote advantage. Thus, when pathogens evolve quickly, the Red Queen is capable of explaining both positive selection and long coalescence times, but the tension between the novel allele advantage and persistence of alleles deserves further investigation.

## Author Summary

Major Histocompatibility Complex (MHC) proteins, responsible for presenting antigens to lymphocytes and thus fundamental to immune response are highly diverse. This diversity, is due to extremely high polymorphism of genes that encode MHC. MHC genes are the paradigm for the positive selection whereby novel MHC alleles are strongly favored by natural selection. This positive selection is widely assumed to be driven by advantage of individuals that are heterozygous at MHC loci and thus are considered to bind wider spectrum of pathogen-derived antigens. In contrast, our simulations showed that novel mutations in MHC are strongly favored by frequency-dependent selection arising from host-pathogen coevolution (the Red Queen process), but not by heterozygote advantage. Our results challenge the predominating view on the processes driving the evolution of MHC genes.

## Introduction

Major histocompatibility complex (MHC) genes code for proteins involved in antigen presentation and initiation of the T-cell-mediated specific immune responses; these genes are the most polymorphic known in vertebrates. Because of its function in pathogen recognition, this high polymorphism is thought to be maintained by some form of balancing selection that is imposed by pathogens. Two mechanisms of balancing selection are most often mentioned: heterozygote advantage, whereby heterozygous individuals gain superior resistance by being able to present a wider range of antigens [1], and Red Queen dynamics, whereby the adaptation of fast-evolving pathogens to the MHC alleles of common host genotypes imposes frequency-dependent selection that favors rare alleles [2]. Additionally, in the latter scenario different MHC alleles may be favored at different times depending on the composition of the pathogen community [3].

Remarkably, the majority of polymorphism at MHC genes is located at the amino-acid residues that encode the Antigen Binding Sites [ABSs; 4,5], consistent with the idea that balancing selection influences the specificity of these residues for different pathogen oligopeptides. At these sites, an excess amount of nonsynonymous substitutions (dN/dS>1), a hallmark of positive selection for amino-acid substitutions, is routinely observed, to the extent that MHC genes have become a paradigm for positive selection [4,6–8].

Both frequency dependence and heterozygote advantage can potentially favor novel alleles that arise by mutation. Such alleles are initially rare, and as such should be favored by negative frequency-dependent selection, but because of the low initial frequency they will occur in heterozygotes. However, the relative roles of these two mechanisms in shaping MHC evolution are far from being understood [reviewed in 9]. Takahata and Nei [10] investigated positive selection under a scenario of overdominance as well as several variants of frequency-dependent selection. These variants included a ‘pathogen adaptation model’, in which novel MHC alleles were assumed to give the host a selective advantage, but the selection coefficient decreased exponentially with time, as well as a ‘minority advantage model’, which assumed that an allele’s fitness was inversely proportional to its frequency. They concluded that, compared to a neutral expectation, the amino-acid substitution rate can be higher, with a longer time to coalescence, under both overdominant selection and frequency-dependent selection resulting from minority advantage, but not under scenario of pathogen adaptation. However, the distinction between the ‘pathogen adaptation model’ and the ‘minority advantage model’ appears to be rather artificial, as both are presumed to result from the same Red Queen dynamics of host-pathogen coevolution. There is thus the need to re-assess the conclusions of Takahata and Nei [10] by allowing the form of selection acting on MHC genes to arise from the nature of their coevolution with pathogens, rather than from arbitrary assumptions about the shapes of fitness functions. Whereas theoretical models based on the nature of MHC-antigen recognition have proven that Red Queen processes are capable of maintaining high levels of MHC polymorphism [see. 11], the role of Red Queen in causing positive selection on the MHC has not yet been investigated.

Here, we take advantage of a modeling framework developed to simulate MHC-pathogen coevolution [11,12], which models ABSs as a string of bits matched to a string of equivalent length representing antigens produced by pathogens. Each pathogen expresses a number of antigens, and each host is exposed to a number of pathogens. These individual-based models do not make assumptions about the strength and shape of selection acting on the MHC, but instead allow for them to emerge as a consequence of host-pathogen interactions. Thus, based on simple mechanisms of MHC-antigen matching, selection favors alleles that produce MHC proteins with a combination of ABS capable of binding the maximal range of antigens produced by pathogens. This simple framework is not meant to be a realistic and exhaustive description of selection acting on the MHC, but rather a vehicle with which to study the consequences of the assumptions we make about the nature of this selection [cf. 13]. Here, we investigate Red Queen dynamics and heterozygote advantage, and determine which is more powerful in retaining new variants in a population and thus capable of explaining the strong signatures of positive selection acting on MHC sequences. To achieve this, we model four scenarios: simultaneous action of Red Queen process and heterozygote advantage (HA+RQ); heterozygote advantage without Red Queen process (HA); Red Queen without heterozygote advantage (RQ); and, as a reference, a scenario lacking pathogens in which only genetic drift acted on hosts (Drift). Our parameter space covers a range of population sizes, as well as three rates of mutations of pathogens: low (2∙10^−4^), intermediate (1∙10^−3^) and high (5∙10^−3^, see Methods for justification). We also examine whether scenarios resulting in stronger positive selection are at the same time capable of maintaining high levels of polymorphism and the long-term persistence of allelic lineages.

## Results

### Pathogen-driven selection and MHC polymorphism

**Fig 1:**
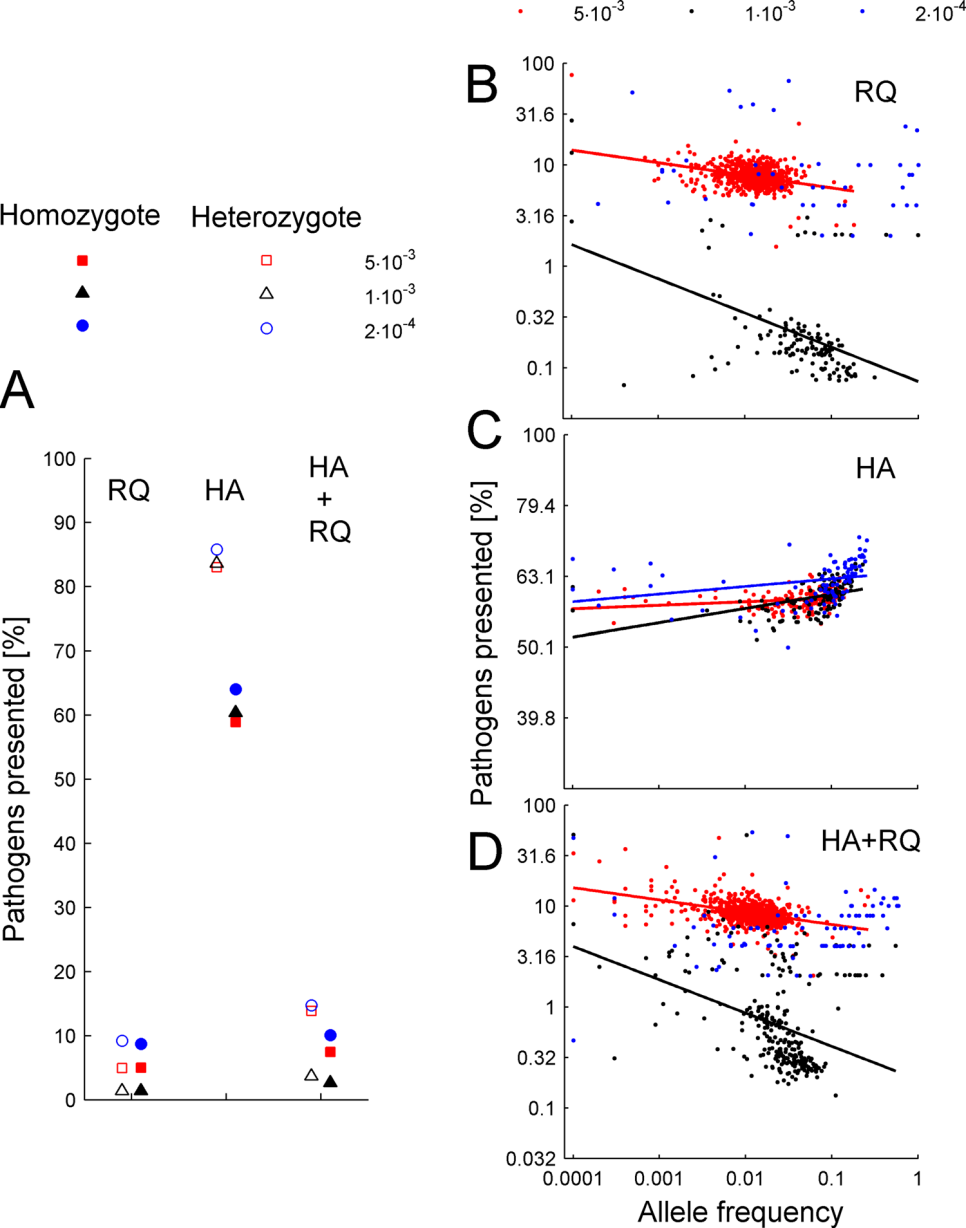
Patterns of frequency dependence and heterozygote advantage that emerged from the model. (A) Comparison of pathogen recognition abilities between heterozygotes and homozygotes, depicted as averages across the last 6000 generations from 10 independent runs. Mutation rates for (A-D) are given in the legends; host population size N = 5000 individuals. (B-D) Relationship between the expected proportion of pathogens recognized by MHC molecules (taken at generation 2000, i.e. at the time when allele numbers stabilized) and allele frequency is shown for three scenarios: Red Queen process (RQ), heterozygote advantage (HA), and both (HA+RQ). Points represent MHC alleles; colors denote pathogen mutation rates (see the legend). Lines represent the statistically significant trends in frequency dependence (see Table A in S2 Appendix).

Both of the types of selection presumed to act on the MHC, heterozygote advantage and frequency-dependent selection, emerged from our model. Heterozygotes had considerably higher fitness than homozygotes when we assumed dominance in the immune response, but not when it was additive (i.e. when the fitness of heterozygotes was a mean of the two alleles) (Fig 1A). Thus, our model successfully eliminated heterozygote advantage while retaining host ploidy (in contrast to Borghans et al. [11], who simulated haploid hosts for that purpose). Interestingly, the advantage was similar (on the order of 50%) in both HA and HA+RQ scenarios, i.e. heterozygote advantage did not depend on whether the frequencies of pathogen haplotypes were shaped by drift or by selection. Similarly, the advantage of heterozygotes did not depend on the pathogen mutation rate (Fig 1A).

We had expected that the Red Queen process would cause negative frequency-dependent selection on MHC alleles. The proportions of pathogens bound by MHC molecules correlated negatively with their frequency under high and intermediate pathogen mutation rates (see Fig 1B and Table A in S2 Appendix). Under the lowest pathogen mutation rate, the trend was negative but not statistically significant (Table A in S2 Appendix). The correlation was the strongest at the intermediate mutation rate (10^−3^), but some common MHC alleles were nevertheless quite successful at pathogen recognition (Fig 1B). This is to be expected under Red Queen dynamics, as resistance-conferring alleles that have recently increased in frequency (such that pathogens have not had time to adapt) should also be present in a pool of common alleles. When the Red Queen process was disabled (Fig 1C) or was weaker than heterozygote advantage because of slow pathogen evolution (Fig 1D, the lowest rate), no negative frequency-dependence was observed.

**Fig 2:**
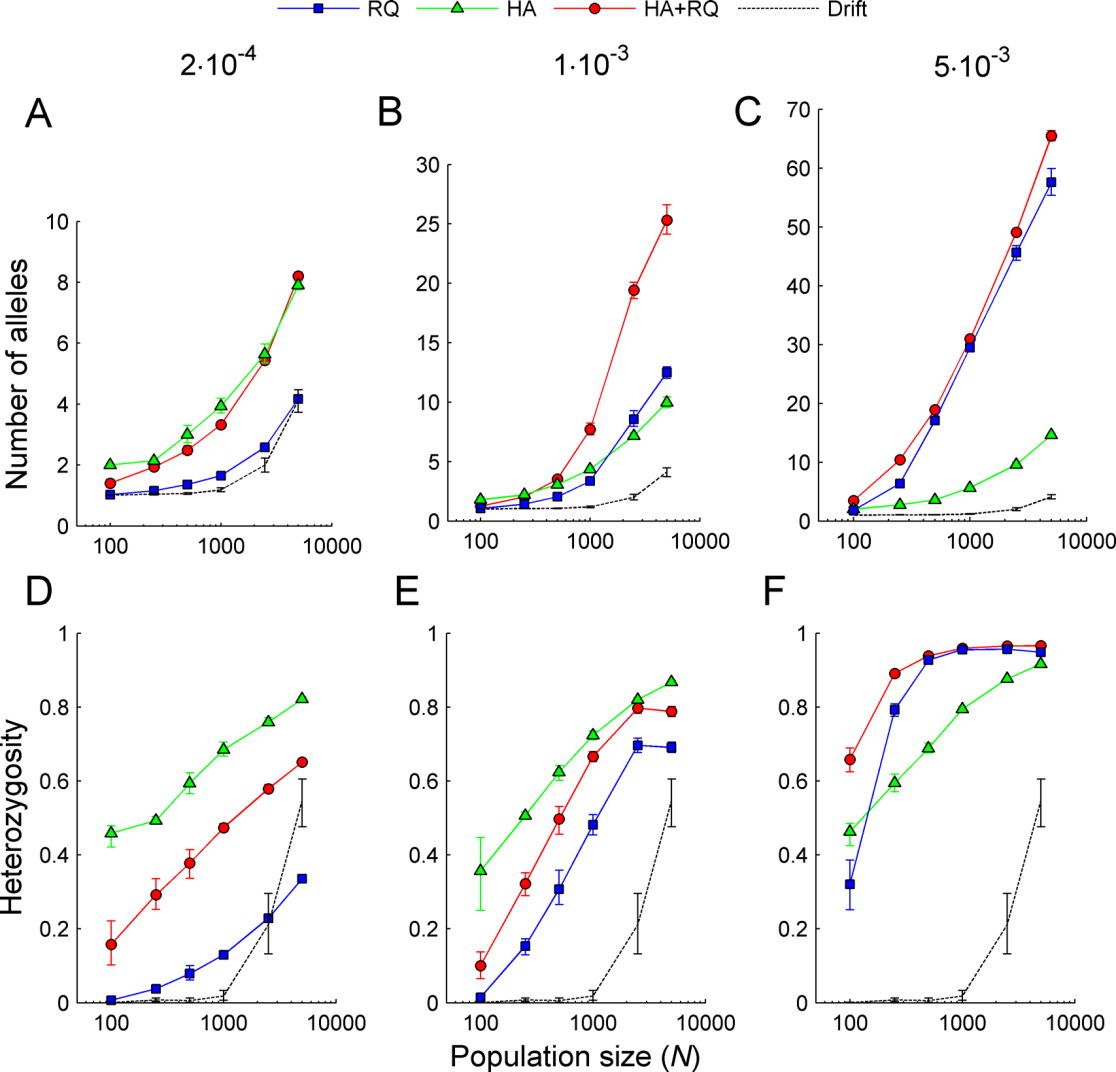
Population size and MHC polymorphism. Impact of population size on the maintenance of MHC polymorphism (upper panels) and heterozygosity (lower panels) under three simulated scenarios–heterozygote advantage (HA), Red Queen (RQ) dynamics, and both (HA+RQ) (see legend)–with three pathogen mutation rates (given above the panels). Data points indicate the mean number of MHC alleles (A-C) and mean heterozygosity (D-F) and are calculated across the last 6000 generations of 10 replicate simulations. Error bars indicate bootstrapped 95% confidence intervals.

Both heterozygote advantage and Red Queen processes were capable of maintaining MHC polymorphism (Fig 2A–2C). MHC polymorphism increased with host population size and with the pathogen mutation rate, but this last effect was much more pronounced under the Red Queen scenario, resulting in much higher levels of polymorphism compared to heterozygote advantage when pathogens evolved quickly (Fig 2A–2C). In contrast, at a low pathogen mutation rate (at which an advantage of rare MHC alleles was not observed, Fig 1B), a higher level of polymorphism was found with heterozygote advantage than with Red Queen dynamics (Fig 2A). At the intermediate mutation rate, heterozygote advantage and the Red Queen process maintained similar numbers of MHC alleles. Interestingly, their effects seemed to combine nearly additively, resulting in a higher number of maintained MHC alleles when they operated simultaneously (Fig 2B). Heterozygosity generally reflected the level of polymorphism, but was also enhanced by heterozygote advantage (Fig 2D–2F).

### Novel allele advantage

**Fig 3:**
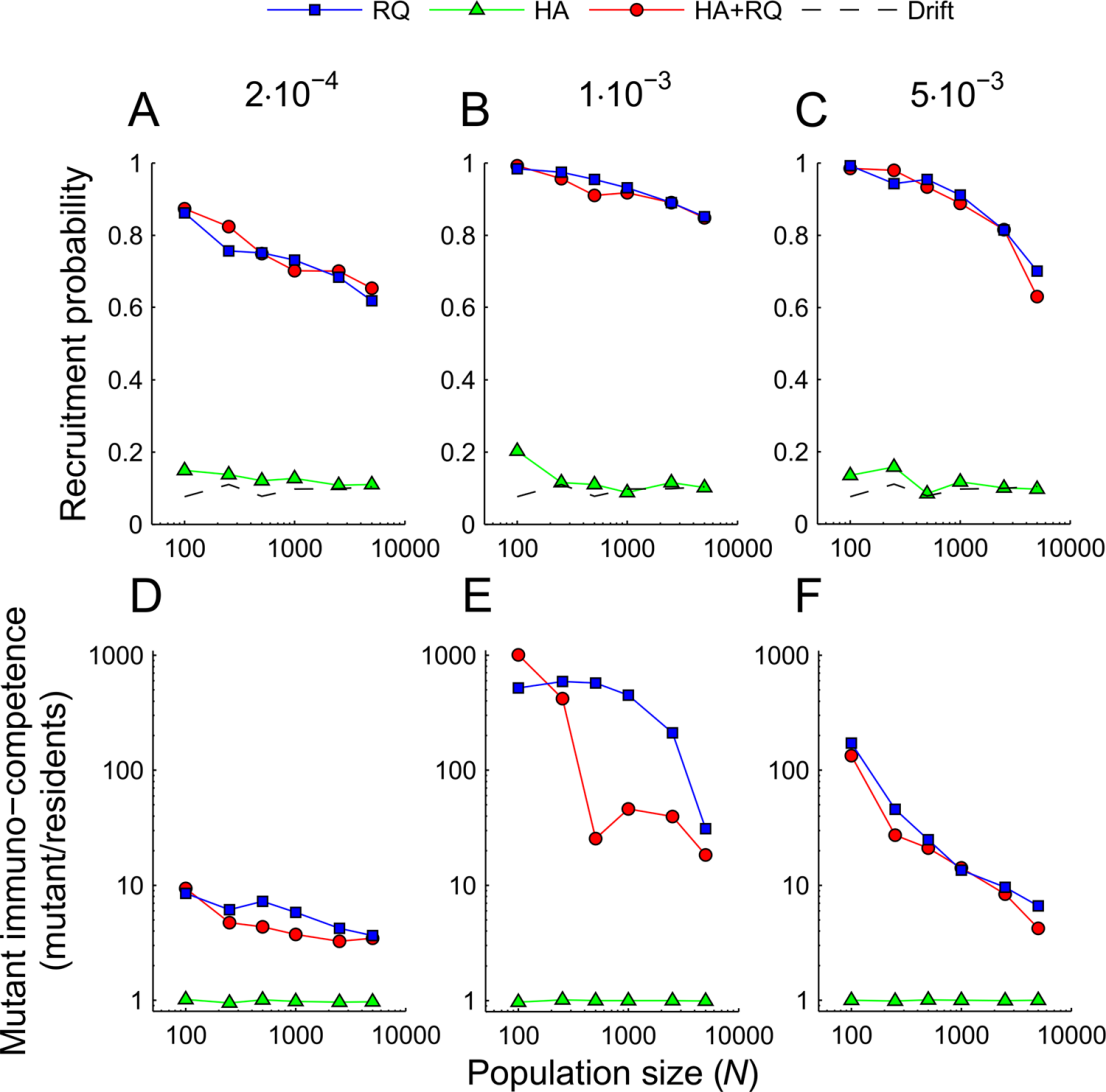
Presentation ability and probability of recruitment of a new mutant MHC allele. (A-C) Data points indicate the probability that a mutant allele will stay in the population for at least 10 host generations (cut-off points longer or shorter than 10 generations (e.g., 2 or 20) showed similar patterns). (D-F) Data points indicate the relative pathogen-recognition ability of a mutant MHC allele relative to the immunocompetence of resident alleles. The average immunocompetence of resident alleles was measured as the number of pathogens recognized in a given generation, weighted by the frequency of each resident allele. Note that a presentation spectrum equal to 1 indicates the threshold at which a mutant allele is able to present, on average, the same proportion of pathogens as resident alleles are. (A-F) Characteristics were calculated across the last 6000 generations of 10 replicates. Scenario labels: HA–heterozygote advantage, RQ–Red Queen, HA+RQ–both. Pathogen mutation rates are given above the panels.

**Fig 4:**
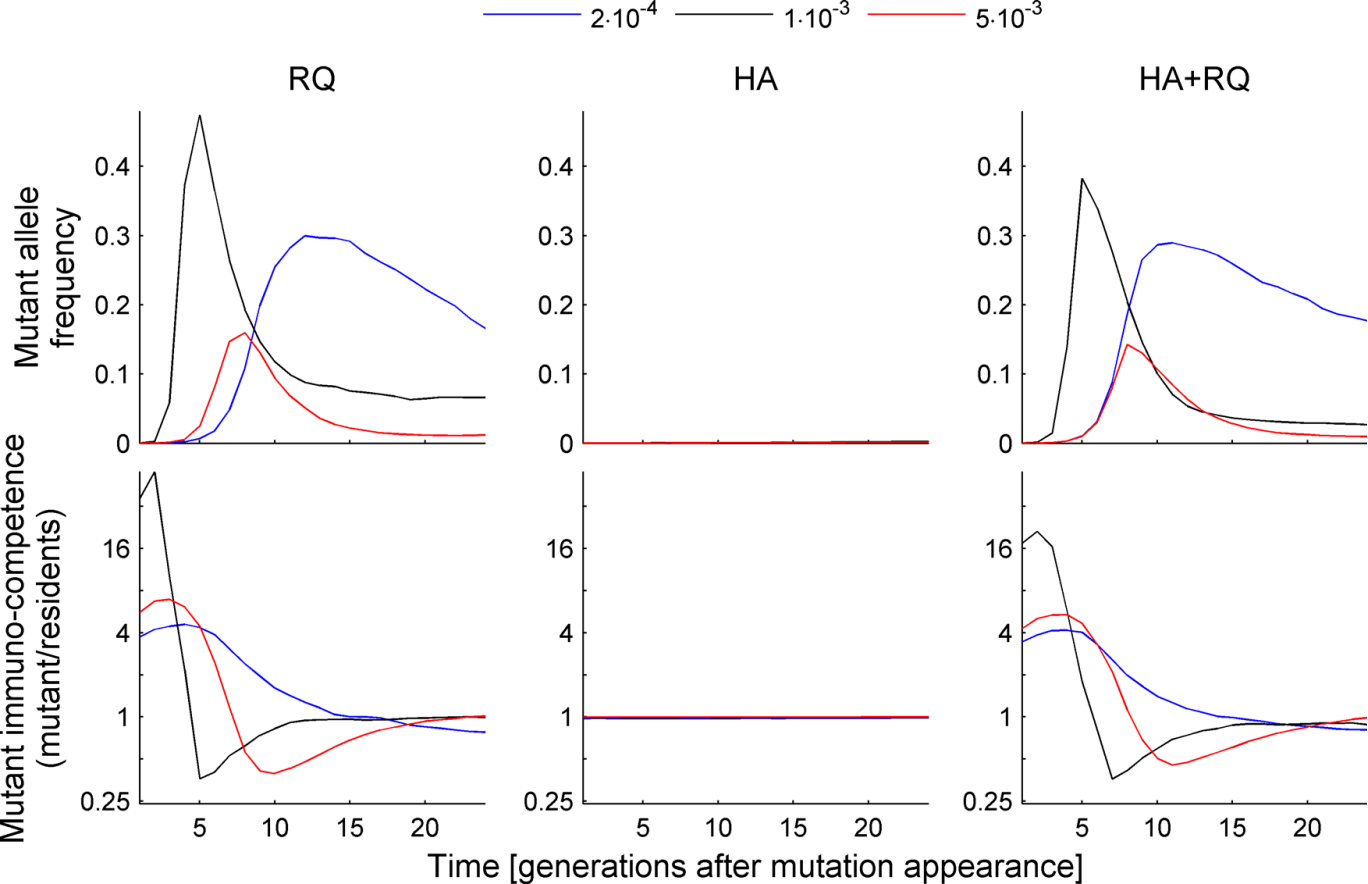
Frequency dynamics and presentation abilities of mutant alleles in the initial period following a mutation’s appearance. Panels in the upper row show averaged allele frequency dynamics over 25 generations after an allele mutation occurred under three mutation rates (see legend). Panels in the bottom row show the dynamics of median mutant allele immunocompetence in relation to the immunocompetence of resident alleles, measured as the number of pathogens presented in a given generation (weighted by the frequency of resident alleles). The measure was calculated across the last 6000 generations of 10 replicate simulations with the host population size N = 5000. Note that a presentation spectrum equal to 1 in the bottom row panels indicate the threshold at which a mutant allele is able to recognize, on average, the same proportion of pathogens as the resident alleles. For presentation purposes the scale on the Y-axis in the bottom row panels was log-transformed. Scenario labels: HA–heterozygote advantage, RQ–Red Queen, HA+RQ–both.

Irrespective of population size or pathogen mutation rate, the probability that a new allele would be retained was much higher under Red Queen scenarios than under heterozygote advantage (Fig 3A–3C). This is because the antigen-presenting capabilities of novel alleles compared to those already segregating in a population were much higher under Red Queen scenarios than under heterozygote advantage (Fig 3D–3F); under the latter conditions, new alleles presented a similar proportion of pathogens as the resident alleles did (Fig 3D–3F). Consequently, under Red Queen scenarios, but not under heterozygote advantage, new alleles usually rapidly increased in frequency over the few first generations (Fig 4). Indeed, for large population sizes (>1000 individuals) the chance of recruitment when only heterozygote advantage was acting (HA) was indistinguishable from drift (Fig 3A–3C). When both forces acted together, the results were similar to the Red Queen process acting alone (Fig 3A–3C). Under Red Queen, but not under the heterozygote advantage scenario, the antigen-presenting capabilities, and consequent probabilities of recruitment of new alleles, decreased with population size (Fig 3). After the initial increase, the frequency of novel alleles decreased until their immunocompetence equaled that of resident alleles (Fig 4, lower panels). Beyond that point, their frequencies fluctuated in a similar way as those of resident alleles (compare [12]).

### Persistence of MHC allelic lineages

**Fig 5:**
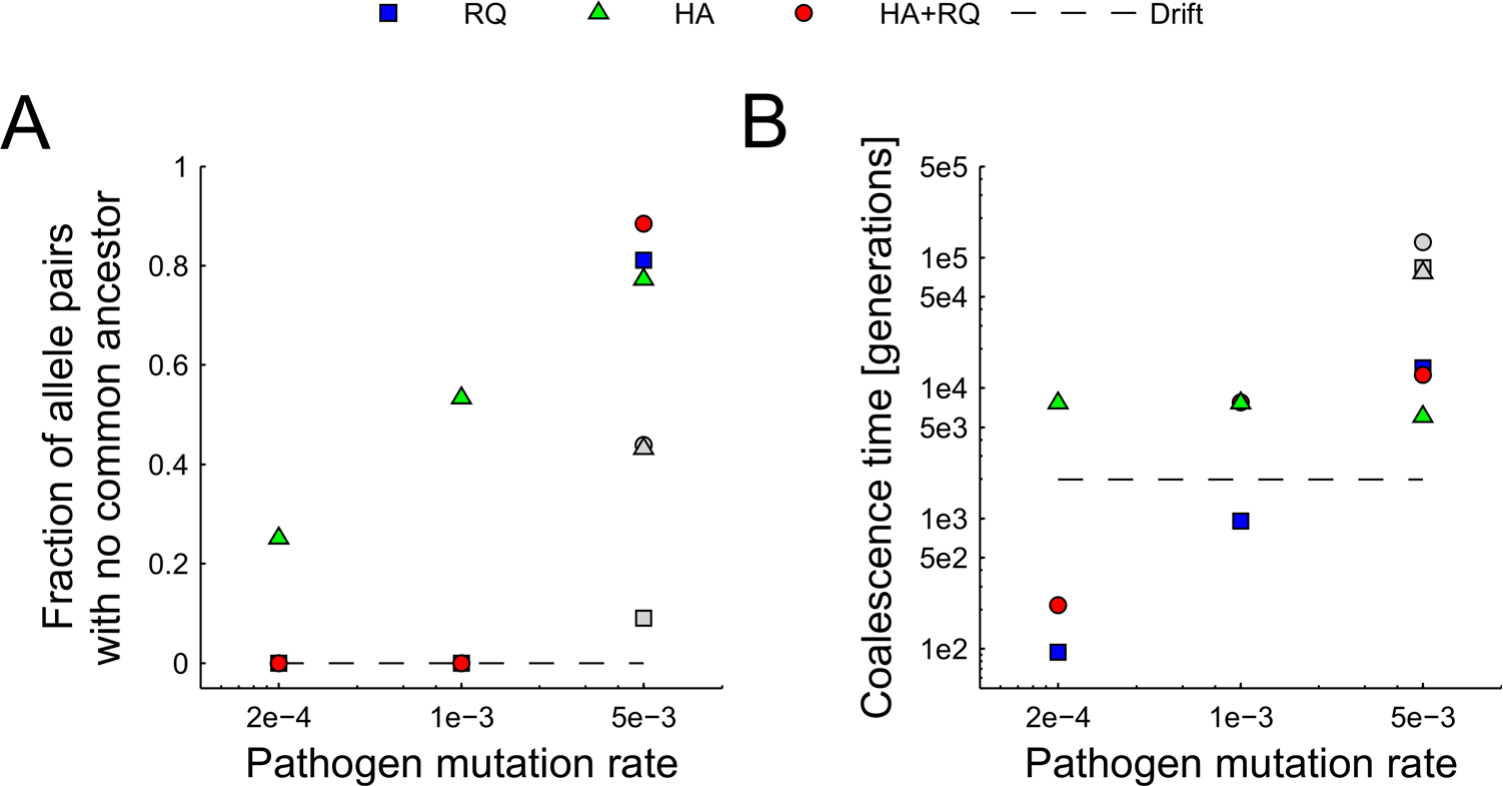
Persistence of MHC allelic lineages. (A) Fraction of allele pairs with no common ancestor within 40 000 generations (red, green, and blue symbols) and 250 000 generations (gray symbols, run only for the highest mutation rate of 5e^-3^) and (B) coalescence times for studied scenarios given in the legend (HA–heterozygote advantage, RQ–Red Queen, HA+RQ–both).

Coalescence times depended on the interaction between the pathogen mutation rate and type of selection. For low and intermediate pathogen mutation rates and under Red Queen dynamics, all allele pairs had a common ancestor within 40 000 generations and no allelic lineage lasted for more than 15 000 generations (Fig 5A and 5B). However, between 25% of alleles (for the lowest mutation rate) and 50% of alleles (under the intermediate pathogen mutation rate) shared no common ancestor when heterozygote advantage acted alone (Fig 5A). In contrast, at the highest mutation rate for all scenarios, over 77% of allelic lineages had no common ancestor (i.e. lasted >40 000 generations). To further explore coalescence times under high mutation rates, we ran these simulations for 250 000 generations and found that 91% of alleles shared a common ancestor when Red Queen processes acted alone, but the average coalescence time for these alleles was quite long: ca. 83 000 generations (Fig 5B). Under heterozygote advantage, or when both processes acted simultaneously, coalescence percentages reached 56.8% and 56.1%, respectively (Fig 5A).

## Discussion

Three features of the evolution of classical MHC genes–a high level of polymorphism, a high probability of retention of amino-acid substitutions in antigen-binding sites, and the long persistence of allelic lineages, resulting in trans-species polymorphism–have been assumed to result from the forces of pathogen-driven balancing selection acting on MHC molecules [4,14]. However, the nature of this selection remains unclear [reviewed in 8,9]. In this respect, our analyses provided two important insights. Firstly, they indicate that the Red Queen process, previously shown to be capable of maintaining high levels of polymorphism [11], is much more likely to drive positive selection on the MHC than heterozygote advantage is, which contrasts with the conclusions of Takahata and Nei [10]. Below, we discuss possible reasons for the disparity between the conclusions of our study and this previous work. Secondly, our results suggest that trans-species polymorphism and positive selection are unlikely to result from the same selective force, because for most parameter combinations the Red Queen process by itself is not capable of maintaining allelic lineages for a longer time than expected under neutrality. We discuss possible alternative scenarios below.

The computer simulations of Takahata and Nei [10] pointed to heterozygote advantage as the force behind both positive selection and the long persistence of MHC allelic lineages. However, both Borghans et al. [11] and De Boer et al. [15] questioned the idea that heterozygote advantage is capable of maintaining the levels of polymorphism observed in natural populations. In particular, De Boer et al. [15] showed that Takahata and Nei’s model was based on the unrealistic assumption that all heterozygotes achieve exactly the same fitness irrespective of the antigen-binding properties of their constituent alleles. When this assumption is dropped, heterozygote advantage can maintain high levels of polymorphism only if the fitness values of different MHC alleles are nearly identical, which is arguably unrealistic. Likewise, simulations by Borghans et al. [11], in which host fitness emerged from the dynamics of simulated host-pathogen interactions, also showed that the number of MHC alleles retained under heterozygote advantage is lower compared to that found in a scenario that allows for host-pathogens coevolution.

Our simulations replicated the main results of the model of Borghans et al. [11]: both heterozygote advantage and Red Queen processes were capable of maintaining a level of MHC polymorphism that increased with host population size and with the pathogen mutation rate. However, our method of manipulating heterozygote advantage (without the need to change ploidy as in Borghans et al. [11]) enabled more direct comparisons between Red Queen and heterozygote advantage scenarios. We found that MHC polymorphism was higher under heterozygote advantage than under Red Queen scenarios at the low pathogen evolution rate; however, consistent with Borghans et al. [11], this degree of polymorphism was still lower than that typically observed in natural populations. At this low mutation rate, pathogens apparently did not evolve fast enough to easily evade recognition by the most common MHC types, as revealed by the flat relationship between an MHC allele’s frequency and its antigen-presenting abilities, and by the relatively high proportion of pathogens recognized by hosts (Fig 1). At the highest mutation rate, Red Queen forces were much stronger in driving MHC polymorphism, yielding numbers of MHC alleles that matched those observed in natural populations. Our simulations thus indicated that the Red Queen process drives the evolution of MHC polymorphism when pathogens evolve quickly, and the heterozygote advantage contributes to its maintenance when the discrepancy of host and pathogen evolution rate is low. We have also found that under an intermediate mutation rate, the joint action of Red Queen forces and heterozygote advantage may maintain higher levels of polymorphism than each of these mechanisms separately. Such an additive effect has not been reported in earlier studies.

Crucially, our results show that heterozygote advantage is much less likely to retain novel MHC variants than the Red Queen process, an aspect not investigated by Borghans et al. [11]. Indeed, the ability of pathogens to adapt to the currently prevailing host genotypes appears to be essential in providing an advantage to novel MHC alleles, which leaves a signature of the excess of non-synonymous over synonymous substitutions [14]. Heterozygote advantage is often assumed to have a similar effect because each novel allele will initially occur in heterozygotes only, but in our simulations this effect turned out to be negligible. The likely reason is that, despite heterozygotes’ having about a 50% advantage over homozygotes in terms of pathogen-recognition potential (Fig 1A), the fact that MHC polymorphism is so considerable implies that most resident alleles, in particular in large populations, occur in heterozygotes (Fig 2D–2F). Indeed, the retention of novel variants was significantly higher under heterozygote advantage compared to drift scenarios only when population sizes were low; such populations had relatively lower levels of heterozygosity (Fig 2D–2F). These population-size-related effects might explain why our conclusions differ from those obtained by Takahata and Nei [10], who studied populations of less than 200 individuals. Furthermore, those authors started their simulations from a monomorphic population, and recorded population history for the subsequent 10 000 generations. Thus, heterozygosity was low early in simulations, which facilitated positive selection under overdominance. This scenario appears to bear little relevance to natural populations, as it would require extreme population bottlenecks during speciation, which is inconsistent with observations of widespread trans-species polymorphism.

It is also worth considering that our study, as well as earlier work [10,11], considered only a single MHC locus, whereas several loci are expressed in most vertebrates [16]. This choice is in some sense justified, because divergent loci are thought to deal with different classes of pathogens [17], and thus to some extent should evolve independently. However, if loci overlap in the pathogen classes that they recognize, the role of heterozygote advantage should further decrease, as heterozygosity would make less of a difference to the range of recognized pathogens in individuals that already bear several alleles (at different loci). Thus, the role of heterozygote advantage in causing positive selection on, and maintaining polymorphism at the MHC may be even less important than our results suggest.

In our simulations, the advantage of novel alleles was modulated by host population size and by the pathogen mutation rate. To our knowledge, this inverse relationship between population size and allele recruitment probability (Fig 3) has not been reported before. The possible explanation for this effect is that both high population sizes and high mutation rates promote high polymorphism of MHC alleles (Fig 2A–2C). As a consequence it is more difficult for new alleles to be highly dissimilar from resident alleles. Furthermore, high polymorphism probably precluded the evolution of ‘generalist’ pathogens which were adapted to most of the MHC alleles segregating in the population. This resulted in a relatively lower advantage for new MHC alleles (Fig 3D–3F). The relationship between the antigen-presenting ability of novel alleles (and in consequence the probability of their retention in populations) and the mutation rate was more complex, as the former was the highest at the intermediate mutation rate. The decline at the highest mutation rate can be explained in the same way as in the case of large populations, i.e. as a by-product of high polymorphism associated with a high mutation rate (Fig 2A–2C). At the lowest mutation rate, on the other hand, pathogens probably could not evolve fast enough to adapt to the resident MHC alleles, again resulting in less of an advantage for novel alleles.

Our results, however, show that the Red Queen process, compared with heterozygote advantage, is much less likely to maintain MHC allelic lineages for a long time, except for at the highest pathogen mutation rate. In this, our results are consistent with those obtained by Takahata and Nei [10], who showed that the advantage of novel alleles (in their ‘pathogen coevolution’ variant) increases the allele turnover rate, and thus considerably decreases coalescence time. In our simulations, Red Queen scenarios had shorter coalescence times than drift under both low and intermediate pathogen mutation rates. Heterozygote advantage, which under these circumstances is capable on its own of both maintaining allelic lineages long-term and affecting the level of MHC polymorphism, had no effect on coalescence times when both processes acted simultaneously. This complete domination by Red Queen dynamics reflects the fact that the probability of a novel allele entering the population under these conditions is virtually identical irrespective of whether heterozygote advantage is acting or not.

Our results thus appear somewhat paradoxical: positive selection, a prevalent feature of the evolution of MHC genes, can be explained by host-pathogen coevolution but not by heterozygote advantage. However, in the Red Queen scenarios, MHC alleles could persist long-term only under a limited range of conditions, specifically, under a high pathogen mutation rate. Interestingly, though, this high mutation rate also produced levels of MHC polymorphism that were most compatible with those observed across many species, so it can be argued that these conditions better approximate those found in natural populations.

Nevertheless, the tension between the degree of novel-allele advantage, which facilitates positive selection, and the persistence of allelic lineages deserves further scrutiny. It is possible that mechanisms other than those resulting from pathogen pressure may contribute to the long-term maintenance of allelic lineages, even in the face of the strong novel-allele advantage due to the Red Queen process. For example, this phenomenon may be better explained by sheltered load of deleterious mutations accumulated in the MHC region [Associative Balancing Complex evolution, 18] or of mate choice [19]. Indeed, the former has been shown to be capable of producing genealogies reminiscent of observed trans-species polymorphism [18]. It also cannot be excluded that trans-species polymorphisms may result from the facilitated introgression of MHC alleles, in addition to (or even instead of) inheritance from common ancestor. For example, Nadachowska-Brzyska et al. [20] documented the facilitated introgression of MHC alleles in two genetically well-separated newt species which diverged ca. 3 mln years ago [21]. Such introgression would be facilitated by positive selection acting on ‘novel’ variants from related species, in which case the Red Queen process would be capable of explaining both positive selection and trans-species polymorphism. Recent reports of MHC introgression in other species [22,23] suggest that this possibility should be seriously considered.

## Materials and Methods

The simulation model of MHC evolution used in this study is based on that of Borghans et al. [11] and further developed by Ejsmond and colleagues [12,19,24]. These models reflect the principle of host-pathogen coevolution that is based on matching the antigen-binding sites of MHC molecules to sequences of antigens produced by pathogens. As stressed in the introduction, they are not meant to be exact mechanistic descriptions of these interactions, but rather reflect their most general features. Consequently, while parameters in these models were chosen to roughly reflect those empirically determined, the exact values of these parameters are not of primary importance to the results. Rather, it was crucial to set the parameter space in such a way as to produce the dynamics of host-pathogen interactions as envisaged by verbal models.

Thus, we consider here sexually reproducing diploid hosts in populations ranging in size from N = 100 to N = 5000 individuals. Each MHC molecule carried by a host is represented by a 16-bit binary string. Each bit may be thought of as a representation of the amino acids that form pockets implicated in the specificity of binding; there are 12–23 polymorphic sites contacting antigens in human MHC molecules [25]. Hosts co-evolve with 50 haploid pathogen species, which to simplify simulations had population sizes equal to that of their hosts (instead of simulating larger pathogen populations, higher probability of mutation occurring in a larger population was emulated by varying pathogen mutation rate), but much shorter generation times (as is typically found in real populations): here, ten pathogen generations per one host generation. Each individual host was attacked by one randomly drawn individual of each pathogen species. Each pathogen produced 20 antigens, represented by 16-bit binary strings. When at least seven bits of an antigen sequence matched an MHC molecule, the pathogen was recognized by the host’s immune system and failed to infect the host (see Fig A in S2 Appendix). These values follow the assumptions of previous models [11,12,24], and thus make our results easily comparable to earlier work. However, they also generate numbers of antigenic motifs available for recognition by a host that are comparable to empirical estimates. For example, our assumptions imply that there are 10 7-bp strings that could be recognized by a host in each antigen, giving a total number of 200 7-bp motifs available for recognition, a value comparable to the number of human influenza virus epitopes that are capable of eliciting an immune response [26]. Consequently, a random MHC molecule had a probability equal to 0.043 of presenting one randomly generated antigen, which is close to empirical estimates [27]. The number of antigens recognized simultaneously did not affect the strength of the immune response and hence had no influence on the simulation results.

In each new generation, each individual host was exposed to one pathogen from each of 50 species. The next generation of pathogens was drawn proportionally to the fitness of each haplotype, i.e. the number of infected hosts. For simplicity, we assumed that a host could be infected repeatedly by the same pathogen species, which is not an unrealistic scenario as multiple-strain infections appear common in nature and often lead to increased virulence [28]. After every 10 pathogen generations, hosts reproduced and the fitness of the hosts was calculated as the proportion of pathogens presented in a given generation. Consequently, hosts that recognized the same number of pathogens, regardless of species, had the same fitness.

### Mutations of hosts’ MHC

In our previous models [12,19,24], mutant alleles were assumed to arise through a micro-recombination process, which was simulated by generating an entire new string of bits [11]. However, here we simulate new MHC alleles arising through point mutations, because positive selection is inferred when there is an excess of amino-acid substitutions at single sites, and changing more than one site simultaneously could make interpretation of the results more difficult. Regardless, simulations that assumed macro-mutations in hosts showed the same patterns as presented in this paper. Similarly to previous models, the rate of mutation per amino acid in hosts did not affect the results as long as it was maintained in similar proportions to the pathogen mutation rate (which we manipulated in the model). To facilitate comparisons with the results of our earlier work we set the rate of mutation per amino acid per host generation to *p*_*t*_ = 6.25∙10^−7^, so that the expected number of new MHC molecules arising through mutation in a host population per generation was the same in this study and our earlier work (assumed to be 10^−5^ per MHC molecule, see S1 Appendix).

### Mutations in pathogen antigens

Haploid pathogens reproduce asexually, such that the genotypes of the offspring and parents were identical except for mutations. We simulated random point mutations in pathogens, i.e. each bit of an antigen could change to the reverse in each generation. We derived populations using three different pathogen mutation rates: 2∙10^−4^, 1∙10^−3^, or 5∙10^−3^ per antigen per pathogen generation (further referred to also as low, intermediate, and high mutation rates). We chose these rates because they were effective in exerting selection on host genotypes (see Fig B in S2 Appendix). At rates lower than those simulated in our study (for instance 1∙10^−4^), pathogens are unable to adapt to host genotypes fast enough, so that the level of polymorphism is low for both heterozygote advantage and frequency-dependent selection [11]. At rates higher than those simulated here (e.g., 1∙10^−2^), effectively random pathogen genotypes are produced and the variation in fitness between host genotypes is negligible [see figure 3 in 24]. The three investigated mutation rates resulted in a broad range of dynamics of host-pathogen coevolution (see Results). For example, the relative role of heterozygote advantage compared to frequency dependence in driving MHC polymorphism was more important at the low mutation rate, but less important at the highest mutation rate. For comparison, the influenza virus NS gene mutates at a rate of 1.5∙10^−5^ [29] which gives roughly 6∙10^−4^ per 20aa antigen.

### Considered scenarios

For each of the three considered pathogen mutation rates, we simulated scenarios that included the two primary mechanisms thought to act on the evolution of the MHC: heterozygote advantage and host-pathogen coevolution (Red Queen process), following the framework of Ejsmond et al. [12]. Here we modeled four scenarios: Red Queen process and heterozygote advantage acting together (HA+RQ); heterozygote advantage without Red Queen process (HA); the Red Queen process only (RQ); and, genetic drift as the only forceacting on hosts (Drift). In the heterozygote-advantage scenario, recognition of a pathogen by only one of the two MHC alleles carried by a host resulted in the same fitness benefit as when both alleles recognized the pathogen, i.e. resistance was dominant. To disable heterozygote advantage, the fitness of a heterozygous individual who carried a pathogen-recognition allele was set to be equal to half of that of a homozygote who bore the same allele, i.e. resistance was additive. To disable the Red Queen process in the HA scenario, pathogens were drawn into the next generation at random from the pool of parasites present at the beginning of the previous generation, irrespective of whether they infected the host or not; in this way, the pathogens did not have the opportunity to adapt to host genotypes.

For all scenarios, simulations were initiated with populations of hosts and pathogens (or hosts only in the Drift scenario) that contained randomly generated genes and antigens; thus host polymorphism was maximal at the outset (see Table B in S2 Appendix) and then evolved to a lower level that was determined by natural selection. This method shortened the time necessary to derive host populations and produced the same results as if evolution had been initiated from just two host alleles [12,24]. For each of the considered scenarios we derived 10 independent replications. The number of alleles stabilized after 2000 host generations for all studied scenarios and was simulated over the next 6000 host generations, i.e. 60 000 pathogen generations.

To investigate the length of time that allelic lineages were maintained under the various scenarios, we additionally ran long simulations (40 000–250 000 host generations) with populations of 2500 hosts, in which we recorded the history of ancestry for all alleles. Then, starting from the last generation, we traced back the alleles’ history to find the common ancestor for any pair of alleles. We recorded the proportion of such coalescence events, as well as the coalescence time (expressed in number of generations). The analysis was performed for all (ca. 12.5∙10^6^) pairs of alleles in a population. Because these simulations were very time-consuming, we ran three replicates for each scenario for 40 000 host generations. This was enough for many lineages to reach coalescence at the low and intermediate mutation rates (see results), but for the highest mutation rate we continued the simulations for 250 000 host generations.

All simulations were performed with Matlab 7.9 (Mathworks, Natick, Massachusetts, USA) and the the results of our computer simulations are available from the Zenodo open digital repository (accession number 18898; http://dx.doi.org/10.5281/zenodo.18898).

## Supporting Information

S1 Appendix The derivation of host mutation rate in MHC genes. (PDF)

S2 Appendix Supplementary tables and figures. (PDF)
