# Supplementary material for: Red Queen Processes Drive Positive Selection on Major Histocompatibility Complex (MHC) Genes
Source: PLoS Comput Biol. 2015 Nov 24;11(11):e1004627. doi: 10.1371/journal.pcbi.1004627 (PMC4658181; doi:10.1371/journal.pcbi.1004627)
Supplement: S1 Appendix — (PDF) [file pcbi.1004627.s001.pdf]

S1 Appendix. The derivation of host mutation rate in MHC genes.

In our earlier work, mutant alleles were assumed to arise through a micro-recombination process (MM) modeled by generating an entire new string of bits [1-3]. However, in the results presented here we simulated changes in MHC genes that were driven by point mutations (PM). Following Borghans et al. [4], MM was modeled by the generation of a new string of randomly chosen bits with probability p_m, corresponding to the fact that micro-recombination leads to the creation of a functionally new MHC molecule. In the PM scenario, bits mutated independently at rate p_t which corresponded to the independent mutation event of each amino acid of an antigen binding site of an MHC molecule. To compare MM and PM scenarios, we assumed that, in both, the expected number of new MHC molecules arising per host generation through the MM process, n_m, and the number arising from point mutations, n_t, is the same, n_m=n_t. Under MM, the expected number of new alleles per generation, n_m, is given by

(1) $n_m = 2Np_m\left(1-0.5^b\right)$

where N indicates the number of diploid host individuals and b is the number of bits in an MHC molecule. The expression 0.5^b is the probability that a randomly generated peptide is identical to the ancestral peptide. In the case of point mutations, the expected number of new MHC molecules arising through mutation, n_t, is given by

(2) $n_t = 2N\left[1-\left(1-p_t\right)^b\right]$

where the expression $\left(1-p_t\right)^b$ indicates the probability that none of the bits change through mutation and the MHC molecule will be identical in parents and offspring. By substituting n_m=n_t we get

(3) $p_t = 1-\left[1-p_m\left(1-0.5^b\right)\right]^{(1/b)}$

For a mutation rate per peptide p_m=10^{-5} (assumed in our earlier work) and number of bits b=16, the point mutation rate that results in the same expected number of new alleles arising per generation equals p_t=$6.25\cdot10^{-7}$

References

1. Ejsmond MJ, Babik W, Radwan J (2010) MHC allele frequency distributions under parasite-driven selection: A simulation model. Bmc Evolutionary Biology 10.
2. Ejsmond MJ, Radwan J (2011) MHC diversity in bottlenecked populations: a simulation model. Conservation Genetics 12: 129-137.
3. Ejsmond MJ, Radwan J, Wilson AB (2014) Sexual selection and the evolutionary dynamics of the major histocompatibility complex. Proceedings of the Royal Society B-Biological Sciences 281.
4. Borghans JAM, Beltman JB, De Boer RJ (2004) MHC polymorphism under host-pathogen coevolution. Immunogenetics 55: 732-739.
