# Supplementary material for: Red Queen Processes Drive Positive Selection on Major Histocompatibility Complex (MHC) Genes
Source: PLoS Comput Biol. 2015 Nov 24;11(11):e1004627. doi: 10.1371/journal.pcbi.1004627 (PMC4658181; doi:10.1371/journal.pcbi.1004627)

S2 Appendix. Supplementary tables and figures.

Table A. The summary statistics for the linear trends between the frequency of MHC alleles and proportion of presented pathogens. The regression lines were fitted to the data derived from simulations of the three investigated scenarios (RQ, HA and HA+ RQ) run under the three pathogen mutation rates (given in the first column). Allele frequencies and proportions of presented pathogens were $\log_{10}$ transformed.

	Slope	R^2
**HA+RQ**		
$2 \cdot 10^{-4}$	0.058	0.03
$1 \cdot 10^{-3}$	**-0.330****	0.14
$5 \cdot 10^{-3}$	**-0.122****	0.17
**HA**		
$2 \cdot 10^{-4}$	**0.011***	0.05
$1 \cdot 10^{-3}$	**0.020****	0.20
$5 \cdot 10^{-3}$	**0.005***	0.08
**RQ**		
$2 \cdot 10^{-4}$	-0.069	0.03
$1 \cdot 10^{-3}$	**-0.337****	0.20
$5 \cdot 10^{-3}$	**-0.124****	0.12

***** p<0.0001, ****** p<0.01

Table B. The mean number of MHC alleles (calculated across 10 independent replications) at the outset of the simulations (first host generation). In the simulations the number of alleles evolved to a lower level that was determined by natural selection (see Fig 2A-C in the main text).

	N=100	N=250	N=500	N=1000	N=2500	N=5000
**HA+RQ**						
$2 \cdot 10^{-4}$	199.5	498.3	991.5	1965.7	4812.4	9272.1
$1 \cdot 10^{-3}$	199.7	498.5	992.9	1969.6	4816.7	9284.0
$5 \cdot 10^{-3}$	199.5	498.3	993.2	1968.7	4814.6	9272.8
**HA**						
$2 \cdot 10^{-4}$	199.2	498.5	990.9	1970.9	4817.7	9273.2
$1 \cdot 10^{-3}$	199.8	498.7	991.7	1970.3	4814.3	9274.9
$5 \cdot 10^{-3}$	199.9	497.3	990.5	1967.4	4815.9	9278.8
**RQ**						
$2 \cdot 10^{-4}$	199.8	497.4	992.5	1970.4	4821.6	9267.5
$1 \cdot 10^{-3}$	199.5	497.6	991.7	1971.0	4815.3	9272.2
$5 \cdot 10^{-3}$	199.8	498.0	991.6	1967.2	4819.3	9273.2

Figure A. An exemplary scheme of the modelled MHC molecules and pathogenic antigens. MHC molecule carried by a host is represented by a 16-bit binary string, similarly to the pathogenic antigens, also represented by 16-bit binary strings. When at least seven adjacent bits of an antigen sequence matched an MHC molecule (indicated by the red rectangle), the pathogen was recognized by the host's immune system and failed to infect the host.

MHC molecule	1 1 0 0 0 1 0 1 1 1 0 0 0 1 1 0
antigen	0 1 0 0 0 1 0 1 0 0 0 1 1 1 1 0

Figure B. Exemplary trajectories showing the variation in host fitness. CV denotes the coefficient of variation for proportion of presented pathogens. The trajectories represent simulations outcome derived under the three investigated scenarios (RQ, HA and HA+RQ) and the three pathogen mutation rates ($5\cdot10^{-3}$, $1\cdot10^{-3}$, or $2\cdot10^{-4}$). The variation in host fitness is presented for populations of 5000 host individuals. Numbers in the panels denote average CV in host fitness calculated across 10 independent replications.

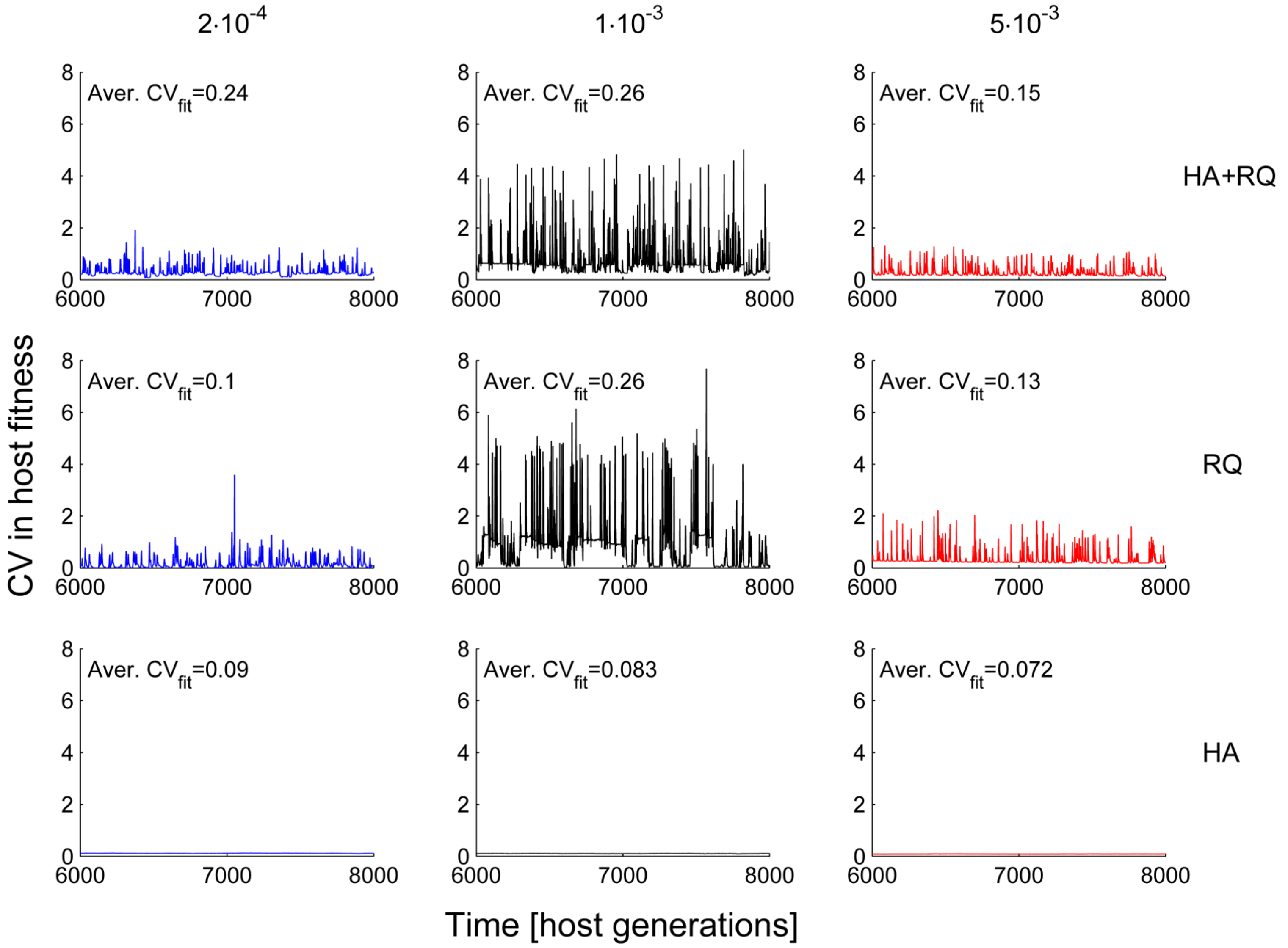

Supplement: S2 Appendix — (PDF) [file pcbi.1004627.s002.pdf]
